# Oblique Deposited Ultra-Thin Silver Films on Polymer Gratings for Sensitive SERS Performance

**DOI:** 10.3390/nano14231871

**Published:** 2024-11-22

**Authors:** Yi-Jun Jen, Meng-Jie Lin

**Affiliations:** Department of Electro-Optical Engineering, National Taipei University of Technology, Taipei 106, Taiwan

**Keywords:** oblique deposition, plasmonic nanostructures, surface-enhanced Raman scattering (SERS), ultra-thin silver films, polymer gratings

## Abstract

A small amount of silver was obliquely deposited onto a polymer subwavelength grating to form a metasurface that comprised silver split-tubes. An ultra-thin silver film with a monitor-controlled thickness of 20 nm at the corner of each ridge of the grating provided the most sensitive surface-enhanced Raman scattering (SERS) measurements. An excitation laser beam that was incident from the substrate provided similar or better SERS enhancement than did the general configuration with the laser beam incident directly on the surface of the nanostructure. Near-field simulations were conducted to model the localized electric field enhancement and to quantify the SERS performance, demonstrating the effectiveness of this novel deposition method.

## 1. Introduction

Surface-enhanced Raman scattering (SERS) is a rapid screening method because it enables the detection of molecular species at the single-molecule level with high sensitivity and speed. It is widely used for food safety, pesticide detection, bioanalysis, chemical analysis, and medical diagnostics [1,2,3,4]. SERS measurements rely on chemical extraction preprocessing of the sample to isolate target molecules, which is critical to achieving sufficient accuracy of detection. The development of substrates that amplify Raman scattering is crucial, as they significantly increase the sensitivity of the SERS technique. The rapid screening and semi-quantitative capabilities of SERS technology play a critical role in risk reduction by enabling preliminary large-scale sample testing before the time-consuming precise assay measurements are made. This method supports the early identification and mitigation of potential risks that are associated with the substances under investigation. The scalability of SERS screening depends on the development of low-cost, highly sensitive substrates. Therefore, devising a method to manufacture SERS substrates cost-effectively and straightforwardly has become an important challenge. Over a decade ago, the manufacturing process of SERS substrates used large amounts of precious noble metals, such as gold and silver, to form the necessary nanostructures, including glancing-angle-deposited nanocluster arrays [5,6], regular arrays of gold nanorods [7], and quasi-3D nanostructure and 2D nanohole arrays that are covered with Au [8].

When light illuminates the surface of the aforementioned metallic nanostructures, localized surface plasmon resonance (LSPR) is enhanced within the nanostructures, forming “hot spots” which beneficially amplify Raman scattering signals [9,10,11,12]. The extensive use of precious metals significantly increases the production costs of SERS substrates, limiting their application and adoption in large markets. Some groups combined a patterned dielectric with a small amount of metal to promote the formation of hot spots in SERS applications. The patterns include metal nanostructured triangular patterns, which yield SERS enhancement factors (EF) at the single-molecule detection level [13]. However, these nanostructures were fabricated using expensive and time-consuming lithographic methods, also limiting the widespread adoption of SERS substrates. Recently, polymer nanopatterns have been produced using a cheap and easy nanoimprinting method [14,15,16,17], which favors the low-cost fabrication of SERS substrates. With the oblique angle deposition (OAD) technique, we have demonstrated the use of a polymer-nanoimprinted grating as a patterned surface: a small amount of silver is obliquely deposited on the ridges of the grating to form a split nanotube array [18,19]. Such an ultra-thin nanotube array has been demonstrated to be a metasurface because the localized magnetic field resonance can be induced by illumination with transverse magnetic (TM)-polarized light with a wavelength in a certain range.

In this study, the silver-coated polymer gratings are prepared with OAD and demonstrated to be sensitive SERS substrates. The composite structure herein comprises only an ultra-thin silver film on each ridge of grating. It only takes a small amount of silver to have a strong SERS signal. Therefore, the thickness of silver on each ridge becomes a critical parameter, and its influence on SERS is investigated. Near-field simulations were performed to assess the localized electric field distributions for different thicknesses of silver on ridges. The silver-coated polymer grating exhibits asymmetrical reflection and absorption properties, allowing SERS measurements with laser illumination on either side of the sample as forward and backward illumination configurations. The near-field simulations were used to compare the electric distributions under forward and backward illumination to explain the experimental result that the SERS signal with backward illumination was stronger than that with forward illumination.

## 2. Experiment

**Figure 1:**
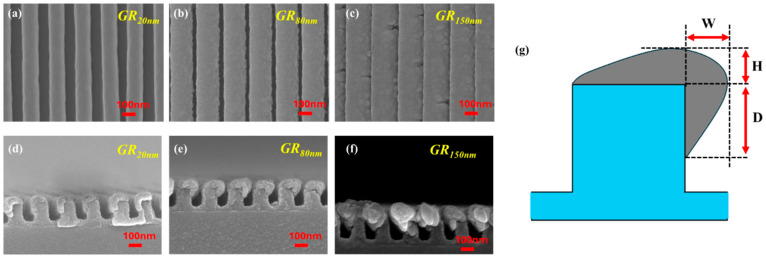
Top-view and cross-sectional SEM images of coated gratings: (**a**,**d**) GR_20nm_*,* (**b**,**e**) GR_80nm_*,* and (**c**,**f**) GR_150nm_; (**g**) schematic diagram of morphology of silver film.

One set of PDAP polymer gratings with pitch lengths of 200 nm were fabricated on BK7 glass as surface-patterned substrates for metal deposition. The ridge height and duty cycle (DC = width/pitch) of each grating were 160 nm and 1/2, respectively. The PDAP nano-grating structures were used as substrates for the oblique deposition of silver. Silver was deposited onto the PDAP gratings using an electron-beam evaporation system. During the oblique deposition, the direction of the grating was perpendicular to the deposition plane, as determined by the normal to the substrate and the deposition flux direction. Deposition was performed in an electron evaporation system, with a deposition angle of *θv* between the substrate normal and the deposition flux direction. The angle *θv* was fixed at 50°. The substrate center was placed 290 mm vertically above the evaporation source. Throughout the deposition process, the substrate temperature was maintained at 10 °C using a water-cooling system. A background pressure of 4 × 10^−6^ was achieved by pre-evacuation before deposition. Quartz monitors were installed next to the rotation stage, allowing the deposition rate of silver to be controlled by a quartz crystal monitor adjacent to the substrate holder. The deposition rate, controlled by the quartz monitor, was 0.3 nm/s. The silver that was deposited under quartz monitor control formed a side-coated grating. Each sample was denoted as, for example, “GR_20nm_”, where the subscripts represent the thickness of the deposited silver. The morphological changes after OAD were analyzed using top-view and cross-sectional scanning electron microscopic (SEM) images of the coated gratings, as shown in Figure 1. Silver was obliquely grown on the top of each grating and on the sides that faced the evaporation source. Owing to shadowing effects, the deposited silver covered one corner of each ridge. The shape of the silver that was deposited on the ridges of the grating resembled shoulders. Therefore, the shape of the deposited silver is specified by the maximum vertical height (H) from the top of the grating ridge, the maximum width (W) from the lateral side of the grating ridge, and the depth (D) of the silver that covered the lateral side of the ridge, as shown in Figure 1g. Table 1 presents the morphological parameters of all samples along with the variances.

## 3. Results and Discussion

### 3.1. Measurement

**Figure 2:**
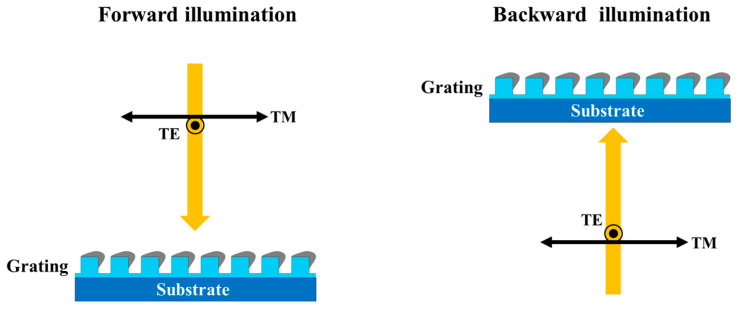
Measurement under forward and backward illumination in the transverse magnetic (TM) and transverse electric (TE) polarized state.

**Figure 3:**
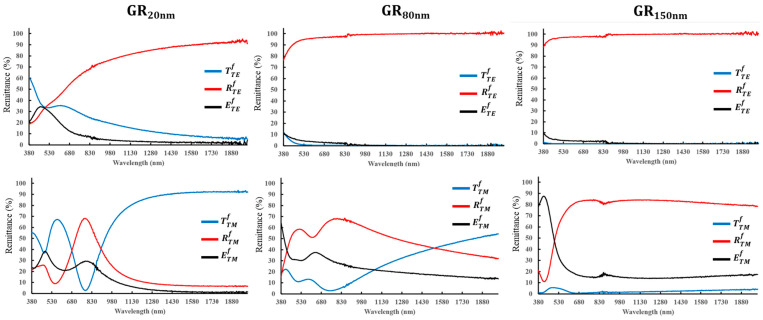
TE and TM polarization spectra of reflectance (R), transmittance (T), and extinctance (E) for each sample under forward illumination.

**Figure 4:**
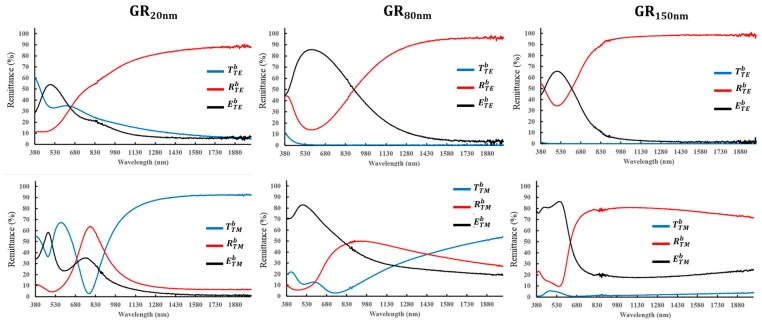
TE and TM polarization spectra of reflectance (R), transmittance (T), and extinctance (E) for each sample under backward illumination.

Figure 2 presents the configuration for spectral measurement under both forward and backward illumination. For the forward illumination, the light wave is incident from the air. Under backward illumination, the light wave is incident from the glass substrate. The direction of the oscillating electric field is perpendicular (parallel) to the grating in the transverse magnetic (TM) and transverse electric (TE) polarized state. The reflectance (R) and transmittance (T) of the samples were measured using a spectrometer (UH4150, Hitachi). The extinctance (E) is given by the relation (E = 1 − R − T). Figure 3 and Figure 4 present the obtained spectra, which are denoted as $R_{j}^{i}$, $T_{TM}^{f}$ and $E_{TM}^{f}$ where the superscript *i* = *f*, *b* indicates forward or backward illumination. The subscript *j* = *TE*, *TM* indicates the polarization state.

As shown in Figure 3, $R_{TE}^{f}$ increases with wavelength from 380 nm to 2000 nm. For GR_20nm_, $R_{TE}^{f}$ increases from 18.5% at a wavelength of 380 nm to 91.2% at 2000 nm. For GR_80nm_, $R_{TE}^{f}$ increases from 76.2% to 90% at short wavelengths from 380 nm to 448 nm, respectively, and exceeds 90% at long wavelengths from 448 nm to 2000 nm. For GR_150nm_, $R_{TE}^{f}$ at long wavelengths from 380 nm to 2000 nm exceeds 90%, but $T_{TM}^{f}$ and $E_{TM}^{f}$ decrease from 10% to 3% as the silver thickness increases from 20 nm to 150 nm.

For TM polarization, the spectra include high reflectance peaks that correspond to localized magnetic field resonance. For GR_20nm_, the highest $R_{TM}^{f}$ peak of 67.6% appears at a wavelength of 785 nm, and the second $R_{TM}^{f}$ peak of 25.7% appears at a wavelength of 465 nm. For GR_80nm_, the wavelength of the highest $R_{TM}^{f}$ peak shifts from 785 nm to 855 nm, and the wavelength of the second $R_{TM}^{f}$ peak shifts from 465 nm to 516 nm. For GR_150nm_, the second $R_{TM}^{f}$ peak disappears, with a high reflectance of 84.1% only at a wavelength of 774 nm. For GR_20nm_, the highest extinctance peak of 38.2% appears at a wavelength of 481 nm, and the second $E_{TM}^{f}$ peak of 29.3% appears at a wavelength of 799 nm. As the silver deposition thickness increases from 20 nm to 150 nm, the highest $E_{TM}^{f}$ peak shifts to a shorter wavelength of 390 nm, and the second $E_{TM}^{f}$ peak disappears.

Figure 4 presents spectra under backward illumination. The $R_{TE}^{b}$ of GR_20nm_ increases from 11.1% at a wavelength of 380 nm to over 80% at 1236 nm. For GR_80nm_, $R_{TE}^{b}$ reaches a minimum at a wavelength of 572 nm. For GR_150nm_, the minimum reflectance shifts to a wavelength of 495 nm. The extinctance under backward illumination exceeds that under forward illumination. The maximum $E_{TE}^{b}$ values of GR_20nm_, GR_80nm_, and GR_150nm_ are 53.9%, 85.6%, and 68% at wavelengths of 498 nm, 572 nm, and 495 nm, respectively.

For TM polarization, the highest $R_{TM}^{b}$ peak of GR_20nm_ is 63.7% at a wavelength of 790 nm. As the silver deposition thickness increases from 20 nm to 150 nm, the highest reflectance peak shifts from 790 nm to 1117 nm and becomes less pronounced. The highest $E_{TM}^{b}$ of GR_20nm_ is 58.3% at a wavelength of 476 nm, and a second $E_{TM}^{b}$ peak at 34.9% is obtained at a wavelength of 739 nm. As the silver deposition thickness increases from 20 nm to 150 nm, the $E_{TM}^{b}$ peak shifts to 86.2% at a wavelength of 551 nm.

### 3.2. Measurement of SERS

**Figure 5:**
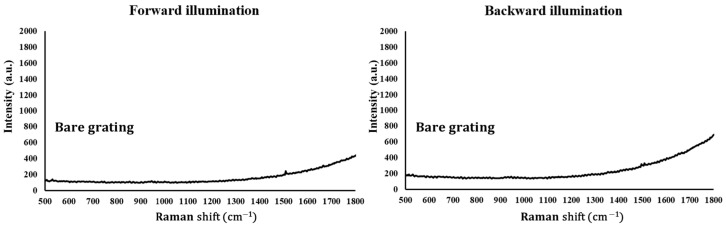
Raman spectra of bare grating for forward and backward illumination.

**Figure 6:**
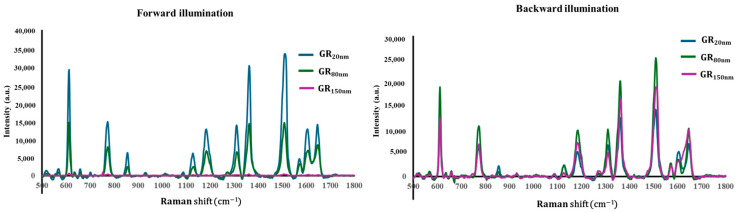
Raman spectra of GR_20nm_, GR_80nm_, and GR_150nm_ for forward and backward illumination.

To make surface-enhanced Raman spectroscopy (SERS) measurements of the prepared grating substrates, rhodamine 6G (R6G) was used as a probing molecule. A 3.5 μL volume of R6G methanol solution with a concentration of $10^{-6}$ M was drop-dispensed onto the surface of the grating substrate, and then allowed to air-dry completely. After the droplets evaporated, they were observed to spread over an area of approximately 5 mm^2^ on each substrate. Raman spectra were obtained using an NS200 Raman spectrometer (NANOSCOPE, Daejeon, Republic of Korea) with an excitation wavelength of 633 nm, a power of 20 mW, a laser spot diameter smaller than 50 μm, and a collection time of 1.5 s. Before saturation adsorption, the SERS signal increased with the concentration of R6G molecules. Figure 5 and Figure 6 present the Raman spectra of the R6G solution adsorbed on bare grating and grating substrates with silver, respectively. Sample measurements were made under both forward and backward illumination. Under forward illumination, the laser beam entered the nanostructure from the air, and the microscopic image on the Raman spectrometer was focused onto the surface of the nanostructure before the measurement mode was switched to Raman spectral measurement. Under backward illumination, the laser beam entered the nanostructure from the glass substrate, and the microscopic image on the Raman spectrometer was focused onto the interface between the glass substrate and the nanostructure before the measurement mode was switched to Raman spectral measurement. The characteristic Raman vibrational peaks of R6G were well resolved at 604, 766, 1379, 1507, and 1668 cm^−1^. The noise spectrum did not exhibit any spectral feature that was related to R6G. Peak signal-to-noise ratios in specific frequency bands were characteristic of R6G. To compare the enhancement effects of the grating substrates, the analytical enhancement factor (AEF) was calculated and analyzed using the formula AEF = (I_SERS_/C_SERS_)/(I_Ref_/C_Ref_), where I_SERS_ and C_SERS_ are the intensity and concentration, respectively, of R6G molecules that are adsorbed on the SERS substrate, and I_Ref_ and C_Ref_ are the intensity and concentration, respectively, of R6G that is adsorbed on the silicon substrate without SERS enhancement.

**Figure 7:**
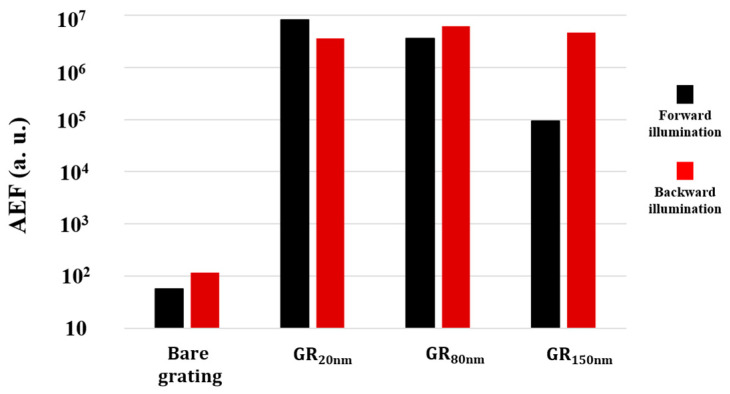
AEF of bare grating, GR_20nm_, GR_80nm_, and GR_150nm_ under forward illumination and backward illumination.

Figure 7 shows the AEFs at 1507 cm⁻^1^ for all grating substrates with respect to an R6G concentration of 10⁻^6^ M. The AEF of the bare grating is only 57 and 118 under the forward and backward illumination, respectively. For GR_20nm_, the AEF value under forward illumination is 8.1 × 10^6^. As the silver deposition thickness changes from 20 nm to 150 nm, the AEF value falls to 9.3 × 10^4^. Under backward illumination, the AEF values of all samples exceed 3 × 10^6^, and reveal similar or higher sensitivity than under forward illumination.

### 3.3. Discussion

**Figure 8:**
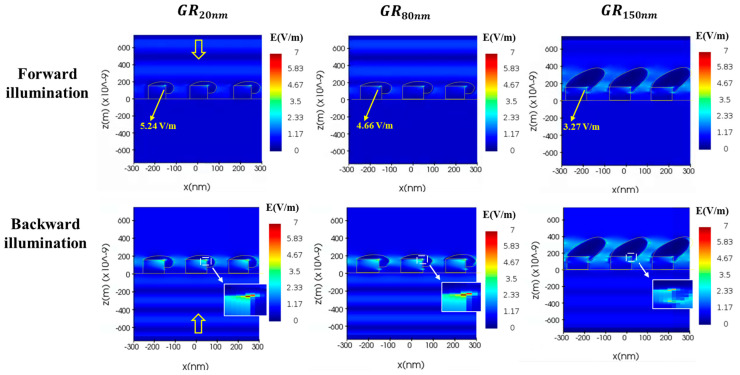
Maximum steady-state amplitude of the electric field in grating substrate under forward and backward illumination.

As shown in Figure 3 and Figure 4, the average extinctance (($E_{TM}^{b}$ + $E_{TE}^{b}$)/2) exceeds (($E_{TM}^{f}$ + $E_{TE}^{f}$)/2), indicating that light-coupling effect is greater than forward illumination when light is incident from the substrate. The strong extinctance explains why the Raman measurements of all samples exhibit high AEF under backward illumination. To elucidate the mechanism of SERS enhancement, finite-difference time-domain (FDTD) simulation (Lumerical Inc., Vancouver, BC, Canada) was conducted to determine the near-field distribution of the electric field amplitude *E*. In the simulations, a harmonic TM plane wave with a wavelength of 633 nm and an amplitude of unity was used as normal illumination, and the size of grid cells was fixed at 5 nm. The refractive index of silver was taken from the database in the Macleod package [20]. The morphology of all samples was taken from the SEM images in Figure 1. Plane electromagnetic waves of unit amplitude propagated from air and glass substrates to the gratings under forward and backward illumination, respectively. Figure 8 plots the variation of the maximum amplitude of the steady-state electric field with the thickness of the silver film. Simulations indicate that a strengthened electric field (hot spot) is present at the corner of the ridge that is covered by the silver film. Under forward illumination, the maximum of the hot spot decreased from 5.46 V/m in GR_20nm_ to 3.27 V/m in GR_150nm_. Under backward illumination, the maximum of the hot spot exceeded 7.98 V/m for all samples. The magnified images in Figure 8 for backward illumination focus on the red areas, highlighting the maximum of the hot spot at the silver-coated corners of the grating’s ridge. The area of the hot spot under backward illumination exceeded that under forward illumination, indicating that backward illumination coupled more energy into the nanostructures.

## 4. Conclusions

The SERS performance of silver films with different thicknesses grown on the top of grating ridges have been investigated in this work. It has been demonstrated that a grating capped with a thinner silver film produces a stronger SERS signal than one with a thicker silver film under forward illumination. Experimental results demonstrate that even a minimal silver-coated grating (GR_20nm_) achieves substantial Raman signal enhancement, approaching an AEF of $10^{7}$ under both forward and backward illumination. The SERS substrate we proposed here can be mass-produced and it is cost-effective in application. On the other hand, the backward illumination configuration allows all optical setup on the backside of the grating and leaves the silver-coated grating facing the analyte. This configuration shows considerable potential for real-time detection applications. In future developments, these enhanced SERS substrates could serve as core components in straightforward, reliable detection tools, advancing sensitive and rapid detection technologies for applications in environmental monitoring and food safety testing.

## Figures and Tables

**Table 1:** Morphological parameters of silver film grown on grating, measured from SEM image.

Sample	GR_20nm_	GR_80nm_	GR_150nm_
H	58.2 ± 4.8 nm	76.8 ± 7.4 nm	115.5 ± 12.5 nm
W	49.2 ± 2.7 nm	91.5 ± 4.4 nm	129.6 ± 11.1 nm
D	89.9 ± 1.4 nm	81.6 ± 1.6 nm	88.8 ± 5.7 nm

## Data Availability

The datasets used and/or analyzed during the current study are available from the corresponding author on reasonable request.
